# The Cytopathic Effect of Different Toxin Concentrations From Different *Clostridioides difficile* Sequence Types Strains in Vero Cells

**DOI:** 10.3389/fmicb.2021.763129

**Published:** 2021-10-12

**Authors:** Zohar Hamo, Maya Azrad, Boris Fichtman, Avi Peretz

**Affiliations:** ^1^The Azrieli Faculty of Medicine, Bar-Ilan University, Safed, Israel; ^2^Clinical Microbiology Laboratory, Baruch Padeh Medical Center, Tiberias, Israel

**Keywords:** *Clostridioides difficile*, multi-locus sequence types, toxin A, toxin B, cytotoxicity effect

## Abstract

*Clostridioides difficile* is one of the leading causes of healthcare-associated diarrhea, with severity ranging from mild, self-limiting disease, to life-threatening toxic megacolon. *C. difficile* infection (CDI) pathogenesis is mediated by the TcdA and TcdB toxins. This work aimed to draw correlations between toxin levels, bacterial strains, and disease severity in 63 CDI patients. *C. difficile* typing was performed by multi-locus sequence types (MLST). Toxin concentrations were measured using the TOX A/B test. In addition, cell cytotoxicity assay was performed following Vero cell exposure to stool extracts (24 h). The most prevalent sequence types (ST) were ST2, ST4, ST6, ST13, ST37, ST42, and ST104, with highest toxin levels produced by ST42 and ST104 (302.5 and 297.1 ng/ml, respectively). These strains had a stronger cytopathic effect (CPE) on Vero cells as compared to strains with lower toxin concentrations (*p* < 0.001), as manifested by lower cell counts and higher percentages of cell rounding and adhesion loss. Although no association was found between ST, toxin concentrations, and disease severity, a diverse *in vitro* effect of different STs on the viability and activity of Vero cells was observed. These findings suggest that disease severity is affected by both host immune responses and by bacterial characteristics.

## Introduction

*Clostridioides difficile* (*C. difficile*) is one of the most prevalent causes of nosocomial diarrhea. It is responsible for approximately 25% of antibiotic-associated diarrhea (AAD) infections and most events of pseudomembranous colitis associated with antibiotic use (Nakao et al., 2020). During the last decade, the epidemiology of *C. difficile* infection (CDI) has changed, with increasing rates of incidence and recurrences. Additionally, the pathogen’s virulence has escalated, presumably due to hyper virulent strains appearance (Stabler et al., 2009). Nevertheless, disease’s manifestations remarkably vary between patients, with some presenting a mild disease and others developing a severe condition.

The main virulence factors contributing to CDI pathogenesis are toxin A (TcdA) and toxin B (TcdB). Following their release, these toxins catalyze the inactivation of small GTPases (such as Rho, Rac, and Ras), which results in cytoskeletal disorganization, hemorrhage, and the release of fluid into the intestinal tract, causing the watery diarrhea that is characteristic of CDI (Voth and Ballard, 2005). Different strains of *C. difficile* can secrete either of the two toxins, both, or neither one. The two toxins are enzymes with glucosyltransferase activity, that inactivates specific Rho and Ras GTPases, and thereby disrupts host cell function (Voth and Ballard, 2005). Toxin B is responsible for induction of RNA synthesis arrest, phagocytosis, immune cell migration, and cytokines’ release. Toxin A causes the accumulation of fluid inside the intestine, while toxin B induces the depolymerization of actin, ultimately damaging the cytoskeleton. Toxin B is thought to have more potent effects compared to Toxin A (Voth and Ballard, 2005).

One of the most virulent strains, ribotype 027/NAP1 *C. difficile*, has a deletion in the *tcdC* locus, which results in increased production of toxins A and B and in increased disease severity (Curry et al., 2007). Some *C. difficile* strains produce a third toxin, the binary toxin, which is found in some epidemic strains such as ribotype 027 and 078 and enhances the severity of CDI disease (Gerding et al., 2014).

*Clostridioides difficile* infection expression varies from diarrhea, to colitis, and, in serious cases, pseudomembranous colitis with toxic mega colon (Evans and Safdar, 2015). While it is clear that disease pathogenesis is affected by the patient’s immune response, that is well-studied, the impact of different strains is still ambiguous. In recent years, there has been an increased interest in differences between *C. difficile* strain characteristics, in order to reveal whether these differences contribute to the versatile disease severity presented by different patients.

Multi-locus sequence typing (MLST) is a new technique for bacterial typing. It detects differences in nucleotides’ sequences of several housekeeping genes. A sequence type (ST) number is given for each strain according to its gene alleles (Maiden et al., 1998). Alignment of all these genes’ sequences from several isolates allow understanding of the evolutionary relationships between the different isolates.

Limited data are available with respect to *C. difficile* STs that are associated with a severe disease compared to those that cause a mild diarrhea or asymptomatic colonization. The few studies that were conducted in this area indicated an association between ST strains and disease severity. Zhou et al. (2014) have reported the ST1 strain to be associated with the greatest disease severity among patients. These data led us to investigate the effect of different *C. difficile* STs (using stool’s extract from different patients) on the morphology and functioning of Vero cells; specifically, we wanted to test whether different strains secrete different levels of toxins and whether the various toxins’ concentration lead to the differences in disease severity.

## Materials and Methods

### Study Population and Sample Collection

Study population consists of patients aged ≥18 years that were hospitalized at the Baruch Padeh Medical Center Poriya, Israel, between November 2017 and February 2020 and diagnosed with CDI as part of the routine patient care services at the Clinical Microbiology Laboratory. A Helsinki Ethics Committee of the hospital (POR-0085-15) approved the study and every patient signed a consent form. All research was performed in accordance with relevant guidelines.

All CDI cases were confirmed by stool examination for toxigenic *C. difficile*, identifying three targets: Toxin B, Binary Toxin, and *tcdC* deletion using the GeneXpert *C. difficile* Polymerase chain reaction (PCR) assay (Cepheid, Sunnyvale, CA, United States).

### Toxin Detection

The presence of toxins A and B was tested using the CerTest *C. difficile* GDH + Toxin A + Toxin B kit (Certest Biotec, S.L, Spain). The kit is based on an immune chromatographic assay for the qualitative detection of *C. difficile* antigen, Glutamate Dehydrogenase (GDH), and toxins A and B from a stool sample. The sample was mixed with a test solution that contains mouse monoclonal antibodies anti-GDH/Toxin A/Toxin B conjugated to red polystyrene latex. When GDH antigen/Toxin A/Toxin B is present in the sample, the antigen/toxin reacts with its specific antibodies in the test solution and this complex is captured by the antibodies in the test strips, resulting in a visible red line.

### Quantification of Toxins’ Concentrations in Stool Specimens

To confirm and calculate the presence and concentration of *C. difficile* toxins, each stool sample was tested by the kit C. Diff TOX A/B IITM, a rapid Enzyme-Linked Immunosorbent Assay (ELISA), according to the manufacturer’s instructions (Tech-Lab, United States). This kit measures the concentration of toxin A and B together. The 96-microassay well plate supplied with the kit contains immobilized affinity-purified polyclonal goat antibodies directed against toxins A and B. Negative control (diluent buffer) and positive control (supplied with the kit) were added to the experiment plate. The assay was performed to determine the concentration of the toxins, directly from fecal extraction. The optical density (OD) was measured at a wavelength of 450 nm on a microplate enzyme-linked immunosorbent assay reader. Quantification of assay results was performed based on a standard curve with known toxin A and B concentrations (ng/ml).

#### Toxin Extracts

Fresh stool (100 g) containing different ST strains were placed in 10 ml of phosphate-buffered saline (PBS), centrifuged at 13,000 × *g* for 5 min, and supernatants were filtered (0.22 μm pore size) in order to extract the toxins. We adjusted all samples to OD_600_ of 1. Diluent (0.2 ml) was added to 0.05 ml of liquid specimen from fecal extraction in order to create a turbidity of 0.5 McFarland in all specimens. Thereafter, the OD was measured at a wavelength of 450 nm, in a microplate enzyme-linked immunosorbent assay reader.

#### DNA Extraction From Stool Sample

Total genomic DNA was extracted using QIAamp DNA Mini Kit (QIAGEN GmbH, Germany). For this purpose, enzymatic lysis buffer was prepared by adding lysozyme (20 mg/ml) to 20 mM Tris⋅Cl, pH 8.0, 2 mM EDTA, and 1.2% Triton. *C. difficile* colonies, grown on CDIF for 48 h, were suspended in 180 μl of the enzymatic lysis buffer, and incubated for 1 h at 37°C. DNA was extracted according to kit’s protocol.

### *Clostridioides difficile* High-Throughput Multi-Locus Sequence Types

The stool samples were cultured on chromID^TM^ *C. difficile* (bioMérieux, Durham, NC, United States) growth medium and then incubated at 37°C in anaerobic conditions for 48 h. *C. difficile* colonies appear asymmetric and black colored. Total genomic DNA was extracted from bacteria using QIAamp DNA Mini Kit (Qiagen, Germany); for this purpose, enzymatic lysis buffer was prepared by adding lysozyme (20 mg/ml) to 20 mM Tris⋅Cl, pH 8.0, 2 mM EDTA, and 1.2% Triton. *C. difficile* colonies, grown on CDIF for 48 h, were suspended in 180 μl of the enzymatic lysis buffer, and incubated for 1 h at 37°C. DNA was extracted according to the kit’s protocol. MLST was performed by setting up Real-time PCR (BioRad CFX96 Real-Time Detection System) and sequencing reactions in 96-well plates (Griffiths et al., 2010). The primers were used to detect seven housekeeping genes (*adk*, *atp*A, *dxr*, *gly*A, *rec*A, *soda*, and *tpi*), using the qPCRBIO SyGreen Blue Mix Hi-ROX kit. After determining the base sequence of the seven housekeeping genes found in each specific strain, the allelic numbers of each gene and the STs were assigned using the PubMLST *C. difficile* database.^1^ The ST number was assigned to each specific combination of alleles. We grouped the isolates according to their ST; each ST group contains at least five isolates. All other STs with less than five isolates were grouped into the “Others” group (ST3, ST8, ST17, ST35, ST43, ST54, ST55, ST88, ST421, and ST439).

### Cytotoxicity Assay

Vero cells, an African green monkey kidney cell line, were purchased from ATCC (CCL81^TM^). Cells were seeded in a six-well plate (3 × 10^5^ cells/well), in Eagle’s Minimum Essential Medium (EMEM), supplemented with 10% fetal bovine serum and supplemented with 5 μg/ml vancomycin and 5 μg/ml streptomycin sulfate (Biological Industries, Beit Haemek, Israel). Cells were grown for 24 h at 37°C, 5% CO_2_ in air atmosphere. One hundred microliters of the final content was added to each well plate and plates were incubated at 37°C, in 5% CO_2_, for 24 h. EMEM mixed with PBS served as negative control. Specimens were considered positive for toxin if the cells in the well were rounded and showed a cytopathic effect (CPE) under a light microscope and scanning electron microscopy (SEM).

### XTT Assay for Determination of Cell Proliferation

Cell proliferation was determined using the XTT [(2-methyloxy-4-nitro-5-sulfophenyl)-2H-tetrazolium-5-carboxanilide]-menadione assay (Tunney et al., 2004), which measures the activity of mitochondrial enzymes such as succinate dehydrogenase (Stone et al., 2009). In this assay, XTT is reduced to a purple formazan by Nicotinamide adenine dinucleotide (H for hydrogen) (NADH), which can be quantified by light absorbance at a specific wavelength. The XTT assay was performed according to manufacturer’s instructions. Briefly, Vero cells were seeded in 96-well microtiter plates (10^4^ cells/well) and incubated for 24 h in an anaerobic workstation. After 24 h, 10 μl of stool extracts with different STs’ toxins strains were added to the wells and allowed to incubate for 24 h under anaerobic conditions.

At this point, 100 μl of XTT was added to each well and incubated in the dark under anaerobic conditions. Readings were taken 30 min following XTT addition, using an ELISA reader (Thermo Fisher Scientific, United States). At least three independent replicates were used for each strain.

### Definitions

Disease severity was defined according to the guidelines of the Society for Healthcare Epidemiology of America and the Infectious Diseases Society of America (IDSA): mild to moderate disease leukocytosis ≤15,000cells/ml and creatinine ≤1.5 times the premorbid level; severe disease leukocytosis ≥15,000 cells/ml or serum creatinine ≥1.5 times the premorbid level. Severe disease was defined also by a serum albumin level of ≤3 g/dl at the time of active infection (Surawicz et al., 2013).

### Scanning Electron Microscopy

To visualize and analyze cells at high resolution, cells were grown at predetermined densities on glass cover slips coated with polylysine. Immediately after treatment with extracts (toxins), cells were prepared as per a previously described protocol (Fichtman et al., 2014), with minor modifications. The specimens were then fixed at room temperature, for 20 min, in Karnovsky’s fixative combined of 2% paraformaldehyde, 2% glutaraldehyde, 80 mM 1,4-Piperazinediethanesulfonic acid (PIPES) buffer, pH 7.4, 50 mM sucrose, and 1 mM MgCl_2_. Fixation and subsequent steps were performed in 6-well or 24-well plates and extra care was taken to avoid even brief drying of the specimens. After the primary fixation, the specimens were washed two times for 3 min in 0.1 M sodium cacodylate (pH 7.4) and post-fixed with 1% osmium tetroxide in 0.1 M sodium cacodylate buffer (pH 7.4) (Electron Microscopy Sciences) for 10 min, at room temperature. Specimens are then washed 2 × 3 min in ultrapure water and dehydrated through a graded ethanol series (3, 7.5, 15, 30, 50, 70, 90, 95, and 100%; 2 × 3 min for each step). Critical point drying was performed from high-purity liquid CO_2_ on a K850 CPD apparatus (Quorum Technologies) and cover slips with cells were mounted on aluminum stubs (Electron Microscopy Sciences) using adhesive carbon tape. In order to increase conductivity of the specimens, the upper surfaces of the cover glasses were bridged with stubs using conductive silver paint (Ted Pella). The specimens were further dried for 30 min under a gentle flow of argon and then coated with a ∼1-nm-thick layer of iridium using a Q150T turbo-pumped sputter coater (Quorum Technologies). Images were acquired with a “Merlin” scanning electron microscope (Zeiss) using in-lens secondary electron detector.

### Statistical Analysis

One-way ANOVA with Welch correction was performed to determine whether fecal toxin concentrations are associated with STs. *t*-test was performed to analyze differences in toxin concentrations between the mild-to-moderate and the severe patient’s groups. All tests applied were two-tailed, and a *p*-value of 5% or less was considered statistically significant. The data were analyzed using SAS^®^ version 9.3 (SAS Institute, Cary, NC, United States).

## Results

### Demographic and Clinical Data

Sixty-three patients with stool specimens positive for *C. difficile* were enrolled in the study (Table 1). The median age of study population was 75.4 years (46–93 years). Twenty-eight (44.4%) patients had a moderate-to-mild disease. Mortality rate following hospitalization was 35%. The majority of CDI cases (73%) were nosocomial.

**TABLE 1:** Demographic characteristics of study’s participants and characteristics of bacterial isolates.

	***N***	**%**
**Gender**		
Female	29	46
Male	34	54
**Toxins**		
A	5	7.9
B	19	30.1
A + B	39	62
**Disease severity**		
Mild-to-moderate	28	44.4
Severe	35	55.6
**Acquired infection**		
Community	17	26.9
Nosocomial	46	73.1
**Mortality**		
Death	22	34.9
Survival	41	65.1

### Bacterial Characteristics

The strains were categorized according to the toxins they produce: 7.9% of the strains produced toxin A, 30.1% produced toxin B, and 62% produced both toxin A and toxin B (Table 1). The binary toxin was not detected in any of the isolates.

**FIGURE 1:**
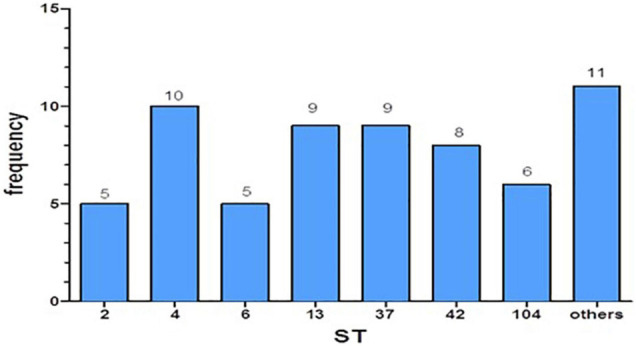
Distribution of the different sequence types (STs) in the study.

Employing the MLST method, we found the ST for each of the isolates. We categorized these strains into seven major ST groups (each group containing at least five isolates) and an additional group called “others,” which combined several different STs (with less than five isolates per ST). The frequencies of the different ST strains were relatively equal (Figure 1). We have not found an association between disease severity and the different STs (*p* = 0.0925).

### Fecal Toxins Concentrations Secreted by the Different Sequence Types

**FIGURE 2:**
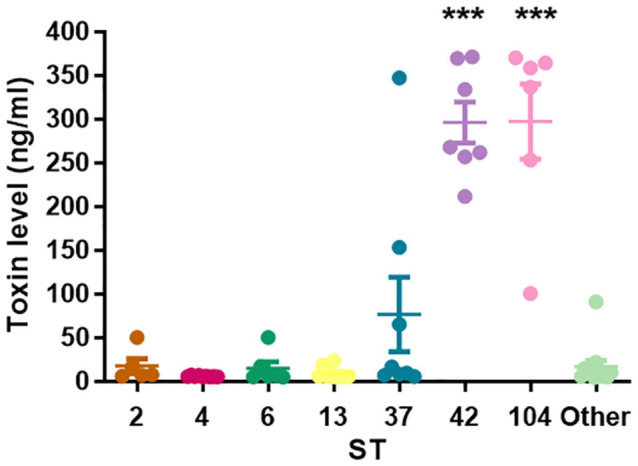
Toxins’ concentrations secreted by different sequence type (ST). The scatter plot showing that ST42 and ST104 were associated with high toxin levels (***p < 0.0001).

We revealed significant differences in toxins’ concentrations among the different STs (Figure 2). In particular, ST42 and ST104 strains secreted significantly higher concentrations of toxins compared to all other groups (*p* < 0.001). Average toxins’ concentrations in positive stool supernatant for ST2, ST4, ST6, ST13, ST37, ST42, ST104, and the “Others” group were 17.31, 5.7, 16.8, 9.2, 37.8, 302.5, 297.1, and 16.8 ng/ml, respectively.

No association was found between toxins’ levels and disease severity (*p* = 0.092); in the mild to moderate disease group, the mean was 55.4 ng/ml (4.83–369.8 ng/ml), and in the severe group, the mean was 99.5 ng/ml (4.06–371.27 ng/ml).

### Cell Proliferation Is Affected by Toxin Concentrations

**FIGURE 3:**
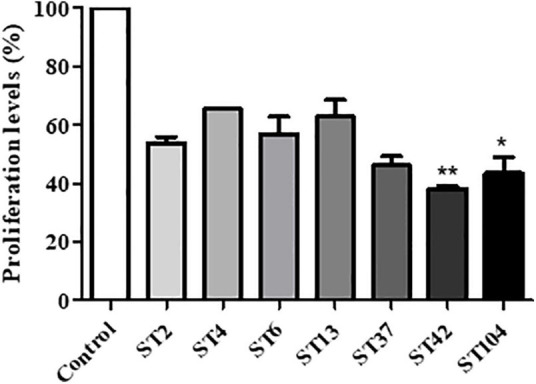
Effect of different sequence type (ST) strains on Vero cells’ proliferation. Vero cells were treated with different ST’s toxins for 24 h. Then, cell proliferation was measured using the XTT assay. *n* = 3 (three different isolates were used for each ST). *p < 0.05 vs. control cells, **p < 0.01 vs. control cells.

In order to examine the effect of different ST’s toxins on cell proliferation, XTT assay was performed on stool extracts with different STs. Exposure of Vero cells to all *C. difficile* strains induced a reduction in cell proliferation, compared to control untreated cells. Among the different STS, ST42, and ST104 that secrete high toxin levels caused the most significant effects, with ST42 causing a 61.8% reduction (*p* < 0.01) and ST104 causing a 56.5% reduction (*p* < 0.05) in cell proliferation, as compared to control cells (Figure 3).

### Increased Fecal Toxin Concentrations Are Associated With More Substantial Cytopathic Effect

**FIGURE 4:**
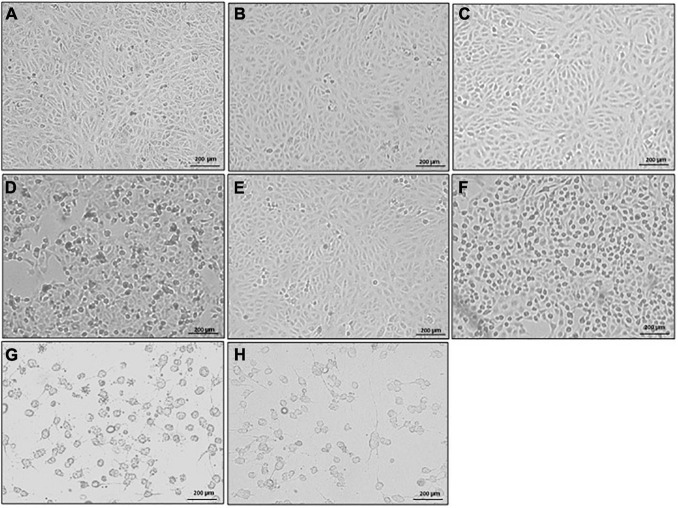
Cytopathic effect (CPE) of different sequence type (ST) strains on Vero cells (light microscope). Stool filtrates were incubated with cells, at 37°C for 24 h. Following incubation, cells were visualized by scanning electron microscope. Representative images of **(A)** Vero cells only, and Vero cells treated with **(B–H)** ST2, ST4, ST6, ST13, ST37, ST42, and ST104-positive stool supernatants, respectively. Scale bar: 200 μm. *n* = 3.

Morphological changes in Vero cells following exposure to stool supernatants with toxins from different STs were observed. The supernatants from the seven ST strains elicited a CPE in Vero cells, with *C. difficile* ST42 and ST104 imparting the highest effects. Cells that were exposed to toxins from ST42 and ST104 were rounded and the number of attached cells was lower, compared to control cells (Figures 4G,H). In contrast, cells that were exposed to ST strains secreting low toxin concentrations (for example ST2, Figure 4B) showed either slight structural changes or definite similarity to the control (Figure 4A).

**FIGURE 5:**
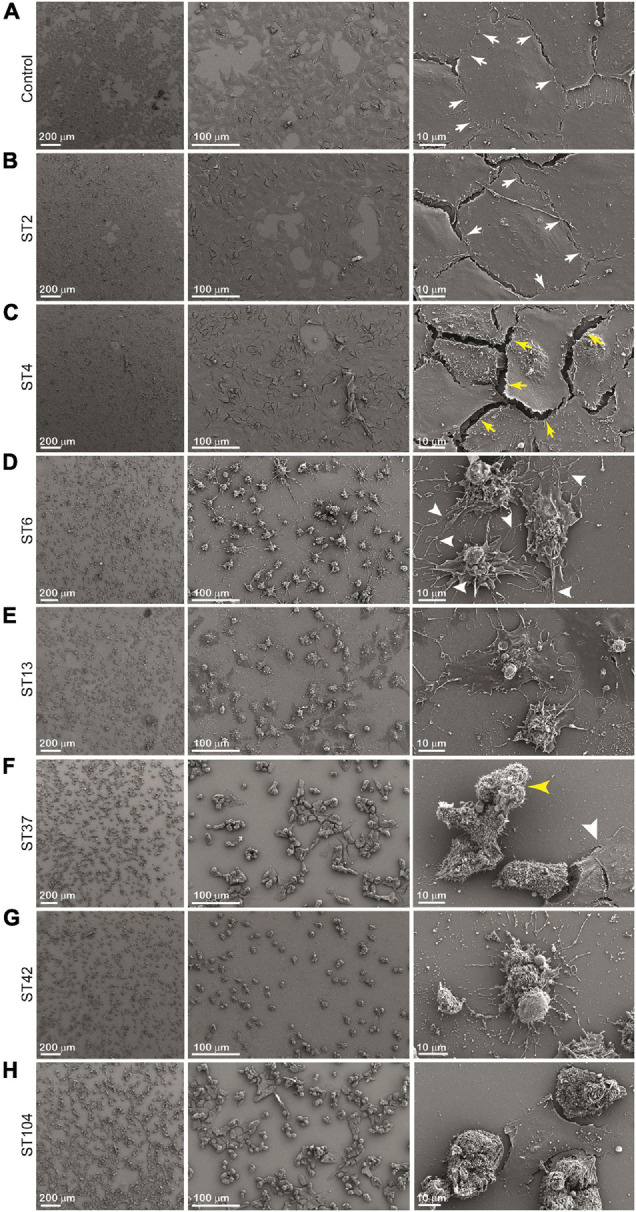
Cytopathic effect (CPE) of different sequence type (ST) strains on Vero cells [scanning electron microscopy (SEM)]. Cells were incubated with stool filtrates at 37°C, for 24 h. Following incubation, cells were visualized by scanning electron microscope. Representative images of **(A)** Vero cells only, and Vero cells treated with **(B–H)** ST2, ST4, ST6, ST13, ST37, ST42, and ST104-positive stool supernatants, respectively. Scale bar: 200, 100, and 10 μm. *n* = 3.

In order to investigate the effect of different stool supernatants on the cells at higher resolution, we used SEM imaging (Figure 5). The results were compatible to those obtained by light microscopy (Figure 4) but in much more detail and with potential to closely examine changes in cellular morphology. Control cells and cells exposed to ST strains secreting low concentration of toxins showed undamaged cell-to-cell adhesion and normally flattened shape (pointing to intact cell-to-matrix adhesion). For example, when we added ST2 stool extract, there was no detectable damage to the cells’ shape and adhesion and treated cells remained very similar to the control (Figures 5A,B). The white arrows point to an intact cell-to-cell adhesion (small gaps between the cells are likely an artifact of sample preparation). Interestingly, treatment of cells with ST4 stool extract, while showing no changes at low magnification (Figures 4C, 5C, left and middle panels), exhibits a very distinguished difference from control at high magnification (Figure 5C, right panel). Yellow arrows point to a large gap between adjacent cells indicating loss of contact. Also, round nuclei are evident, suggesting changes in cell shape, possibly due to partial loss of cell-to-matrix contacts as well.

Extracts from strains ST6, ST13, and ST37 show more dramatic effect evident even at low magnification (Figures 5D–F, left and middle panels). High-magnification images indicate total loss of cell-to-cell contacts as a large gap between cells are present (Figures 5D–F, right panels) while cell density is similar for all conditions as shown in Figures 5A–H, left panels. It is intriguing to observe that high-magnification images show differences between supernatants ST6, ST13, and ST37 on cells morphology. Cells treated with ST6 extract are round and have completely loose cell-to-cell interaction while still having a considerable number of cell-to-matrix adhesion points (Figure 5D, right panel, white arrowheads). However, cells treated with ST13 extract are more flattened in shape, indicating only a mild decrease in cell-to-matrix contacts, although connections between adjacent cells are disrupted completely (Figure 5E, right panel). Between mentioned extracts (ST2, ST4, ST6, ST13, and ST37), ST37 shows a more severe impact on cells’ morphology. After treatment with ST37, cells were observed mainly in lumps with some single round cells. Majority of clamped cells were completely detached from the dish, but still strongly attached to each other, indicating that cell-to-matrix contacts are more severely affected by this strain than cell-to-cell contacts (Figure 5F, middle and right panels). Yellow arrowhead points at claimed round cells that lost contact with matrix, but still strongly attached to each other. The reason for the presence of small amount of less effected “resistant” cells (white arrow head) might be that less affected cell-to-cell contacts restrict accessibility of cell-to-matrix adhesion points for the toxins.

The most dramatic effect on cell morphology was observed in cells that were exposed to ST strains secreting higher concentrations of toxins—ST42 and ST104, which were associated with the greatest morphological changes in cells, including cell rounding and loss of adhesion (Figures 5G,H). Cells exposed to ST42 were all rounded and had very fragile string-like extensions. Majority of the cells were singles with some lumps present, indicating significant damage to both cell-to-cell and cell-to-matrix contacts (Figure 5G, right panel). Notably, despite the same effect on cellular proliferation (Figure 3) and seemingly similar appearance in light microscopy (Figures 4G,H), ST42 and ST104 affected cells differently, as evident from high-magnification SEM images (Figures 5G,H, right panels).

## Discussion

Identifying the role of strain differences in CDI severity may be valuable for treatment adjustment; it is possible that patients with virulent strains should be managed more aggressively than patients with less virulent strains. Since toxins are the main virulence factors of *C. difficile*, we thought to investigate whether different strains secrete distinct levels of toxins, which may explain the versatile severities of disease in patients.

As we assumed, the different strains secreted various concentrations of toxins. To the best of our knowledge, this is the first study that presented toxin levels among different STs. Nevertheless, a previous study in which ribotyping—another strain typing methodology—was used has detected diverse toxins’ yield among different strains (Akerlund et al., 2006).

We did not find an association between strain type and disease severity. Since our study is preliminary and has a small sample size, further investigation should be performed in order to define whether strain type affects disease severity. On the other hand, it is possible that toxin levels’ effects are more restricted to the intestine and disease severity is affected by the multisystemic response of the host. For example, the presence of neutralizing antibodies in different levels may explain why we could not find an association between toxin levels and disease severity.

Surprisingly, no association was found between toxin concentrations and disease severity. Interestingly, a few previous studies have pointed out the association between fecal toxin levels and proxy measures of disease severity (Burdon et al., 1981; Akerlund et al., 2006). For example, Burdon et al. (1981) have correlated high fecal toxin titer with the presence of pseudomembrane in patients; in another study, increased fecal toxin levels were measured in patients with more than 10 loose stools per day, compared to patients with 3–10 and less than 3 loose stool per day. However, when *in vitro* toxin yields were measured directly from *C. difficile* isolates, no correlation was found between toxin levels and disease severity (Akerlund et al., 2006).

In contrast, a previous study in Israel in 2017 showed that fecal *C. difficile* toxin levels were significantly higher in patients with severe disease compared with those with mild to moderate disease (Cohen et al., 2018). It is possible that toxin concentrations alone do not directly affect disease severity, but together with other bacterial characteristics. Thus, further studies should be performed in order to affirm this association.

In relation to this, we could point out a link between ST type, toxin levels, and the changes that the strains inflicted on Vero cells’ viability and morphology. Higher toxin levels were found in stool with *C. difficile* ST42 and ST104, and these strains induced a more aggressive CPE, as compared to the other STs. The morphology of Vero cells that were exposed to ST42 and ST104 changed the most, as manifested by cell rounding, adhesion loss, and the lowest cell number. Therefore, it may be assumed that different STs affect differently the host, and this can be partially explained by the versatile production of toxins.

This study demonstrates, for the first time, differences between the ST strain types in toxin concentration and their effects on cell morphology and activity. High levels of fecal toxins can cause more significant destruction of intestinal cells and rapid deterioration in patients. These findings suggest that disease severity may be affected by both host immune responses and by bacterial characteristics. Since we did not find associations between ST, toxin concentrations, and disease severity, further investigation should be performed in order to understand the contribution of bacterial characteristics and specifically the different toxin concentrations to disease severity. If toxin levels affect disease severity, then measurement of toxin levels will help to adjust treatment according to the severity of disease.

We believe that, over time, we will transition to an adapted medicine for a specific strain of bacterium and not just based on patient characteristics.

## Data Availability Statement

The original contributions presented in the study are included in the article/supplementary material, further inquiries can be directed to the corresponding author.

## Ethics Statement

The studies involving human participants were reviewed and approved by the Helsinki Ethics Committee of the Padeh Poriya Medical Center, POR-0085-15. The patients/participants provided their written informed consent to participate in this study.

## Author Contributions

ZH and AP: conceptualization. ZH, MA, and AP: data curation, investigation, validation, visualization, and writing—original draft. ZH, MA, BF, and AP: formal analysis, methodology, and writing—review and editing. MA and AP: project administration. MA, BF, and AP: supervision. All authors have read and agreed to the published version of the manuscript.

## Conflict of Interest

The authors declare that the research was conducted in the absence of any commercial or financial relationships that could be construed as a potential conflict of interest.

## Publisher’s Note

All claims expressed in this article are solely those of the authors and do not necessarily represent those of their affiliated organizations, or those of the publisher, the editors and the reviewers. Any product that may be evaluated in this article, or claim that may be made by its manufacturer, is not guaranteed or endorsed by the publisher.

## Footnotes

1
http://pubmlst.org/cdifficile/
